# Muckle-Wells Syndrome Across Four Generations in One Czech Family: Natural Course of the Disease

**DOI:** 10.3389/fimmu.2019.00802

**Published:** 2019-04-16

**Authors:** Šárka Fingerhutová, Jana Fráňová, Eva Hlaváčková, Eva Jančová, Leona Procházková, Kamila Beránková, Markéta Tesařová, Eva Honsová, Pavla Doležalová

**Affiliations:** ^1^Paediatric Rheumatology and Autoinflammatory Diseases Unit, Department of Paediatrics and Adolescent Medicine, First Faculty of Medicine, General University Hospital in Prague, Charles University, Prague, Czechia; ^2^Department of Paediatric Rheumatology, University Hospital Brno, Brno, Czechia; ^3^Department of Clinical Immunology and Allergology, St. Anne's University Hospital in Brno and Faculty of Medicine, Masaryk University, Brno, Czechia; ^4^Department of Nephrology, First Faculty of Medicine, General University Hospital in Prague, Charles University, Prague, Czechia; ^5^Department of Rheumatology, St. Anne's University Hospital Brno, Brno, Czechia; ^6^Laboratory for Study of Mitochondrial Disorders, First Faculty of Medicine, General University Hospital in Prague, Charles University, Prague, Czechia; ^7^Clinical and Transplant Pathology Centre, Institute for Clinical and Experimental Medicine, Prague, Czechia

**Keywords:** cryopyrin-associated periodic syndromes (CAPS), cryopyrinopathy, Muckle-Wells syndrome (MWS), familial cold autoinflammatory syndrome (FCAS), AA amyloidosis, hearing loss, rash

## Abstract

**Background:** Muckle-Wells syndrome (MWS) represents a moderate phenotype of cryopyrinopathies. Sensorineural hearing loss and AA amyloidosis belong to the most severe manifestations of uncontrolled disease. Simultaneous discovery of MWS in four generations of one large kindred has enabled us to document natural evolution of untreated disease and their response to targeted therapy.

**Methods:** A retrospective case study, clinical assessment at the time of diagnosis and 2-year prospective follow-up using standardized disease assessments were combined.

**Results:** Collaborative effort of primary care physicians and pediatric and adult specialists led to identification of 11 individuals with MWS within one family. Presence of p.Ala441Val mutation was confirmed. The mildest phenotype of young children suffering with recurrent rash surprised by normal blood tests and absence of fevers. Young adults all presented with fevers, rash, conjunctivitis, and arthralgia/arthritis with raised inflammatory markers. Two patients aged over 50 years suffered with hearing loss and AA amyloidosis. IL-1 blockade induced disease remission in all individuals while hearing mildly improved or remained stable in affected patients as did renal function in one surviving individual with amyloidosis.

**Conclusions:** We have shown that severity of MWS symptoms gradually increased with age toward distinct generation-specific phenotypes. A uniform trajectory of disease evolution has encouraged us to postpone institution of IL-1 blockade in affected oligosymptomatic children. This report illustrates importance of close interdisciplinary collaboration.

## Introduction

Cryopyrinopathies (Cryopyrin Associated Periodic Syndromes, CAPS) belong to autoinflammatory disorders with autosomal dominant inheritance caused by the gain-of-function point mutation of *NLRP3* (NACHT, LRR, and PYD domains-containing protein 3) gene which encodes cryopyrin and leads to interleukin-1 (IL-1) overproduction (1–3). This is reflected in the newly proposed nomenclature of “NLRP3-associated autoinflammatory diseases” (mild, moderate, severe) (4). Estimated population frequency ranges between 1 and 3 per million (2). The spectrum of its clinical presentation is wide from the mildest Familial Cold Autoinflammatory Syndrome (FCAS) across Muckle-Wells Syndrome (MWS) to the most severe Chronic Infantile Neurological Cutaneous Articular (CINCA) syndrome (5). Early onset urticarial rash is a key symptom in all phenotypes (6). Among them, MWS is associated with the high risk of severe complications including hearing loss, vision impairment and AA amyloidosis (7). The latter occurs in as many as 30% of MWS patients (6). Hearing loss initially affecting high sound frequencies may be under recognized until irreversible damage develops (6). It has been shown that various clinical manifestations within the overlapping CAPS spectrum could be associated with specific single mutations with varying penetrance (8). Early diagnosis and appropriate treatment with IL-1 blockade may prevent the development of late complications and improve patients' quality of life (7).

We report a family with multiple members affected with MWS and the way leading to their diagnosis. Analysis of the case histories illustrates the natural disease evolution in untreated individuals with the same genotype across four generations. Prospective systematic follow-up confirms the response of various disease features to IL-1 blockade with anakinra.

## Patients and Methods

Based on the referral of a pediatric patient to the fever clinic of General University Hospital in Prague with a suspected diagnosis of CAPS multiple members of his large family were gradually identified. This has enabled to collect and analyse their past clinical data and follow them prospectively starting from end 2015 for a minimum of 2 years. Written informed consent with clinical data collection was obtained from all patients or their legal guardians as a part of the Eurofever project (No. 2007332) approved by the institutional ethics committee of General University Hospital in Prague. All patients and/or their legal guardians approved publication of their case histories by signing the consent form. Patient history and previous results were retrieved from health records of their general or pediatric practitioners and/or specialist clinics as appropriate. Additionally, an independent detailed interview was carried out by one of the investigators (SF). At the time of referral each patient underwent full clinical examination. Blood tests consisted of inflammatory parameters Erythrocyte Sedimentation Rate (ESR), C-reactive protein (CRP), serum amyloid A (SAA), full blood count (FBC), liver and renal function tests, immunoiglobulins, DNA analysis, and urine analysis (U/A). Additional tests included fundoscopy, neurological examination and audiometry using frequencies of 500, 1,000, 2,000, 4,000, 6,000, and 8,000 Hz for the evaluation of pure-tone air conduction. Chest X-ray, Quantiferon TB Gold, and tuberculin test were performed prior to the initiation of biological therapy.

Patients were followed prospectively at 3-monthly intervals by clinical examination and blood tests. Additionally, they were asked to keep completing an Auto-Inflammatory Disease Activity Index (AIDAI). This is a patient diary recording daily presence of disease manifestations (fever, headaches, musculoskeletal and eye symptoms, skin rash,1 point for each present item) (9). The maximum monthly cumulative score is 403 for CAPS patients (for a 31-day month). Inactive disease has been defined as an AIDAI below 9 (9). In addition, the preliminary version of Autoinflammatory Disease Damage Index (ADDI) (10) was calculated for all patients at the first and the 2-years visit.

Exon 3 of *NLRP3* gene (ENSG00000162711, NM 004895) was amplified by PCR in five overlapping fragments from genomic DNA isolated from leukocytes and analyzed by direct sequencing using 3,500xL genetic analyser (Applied Biosystems, USA). PCR primers are available upon request. Recently, mutations in other exons of *NLRP3* gene were excluded by targeted NGS sequencing (MiSeq, Illumina, USA).

## Results

**Figure 1:**
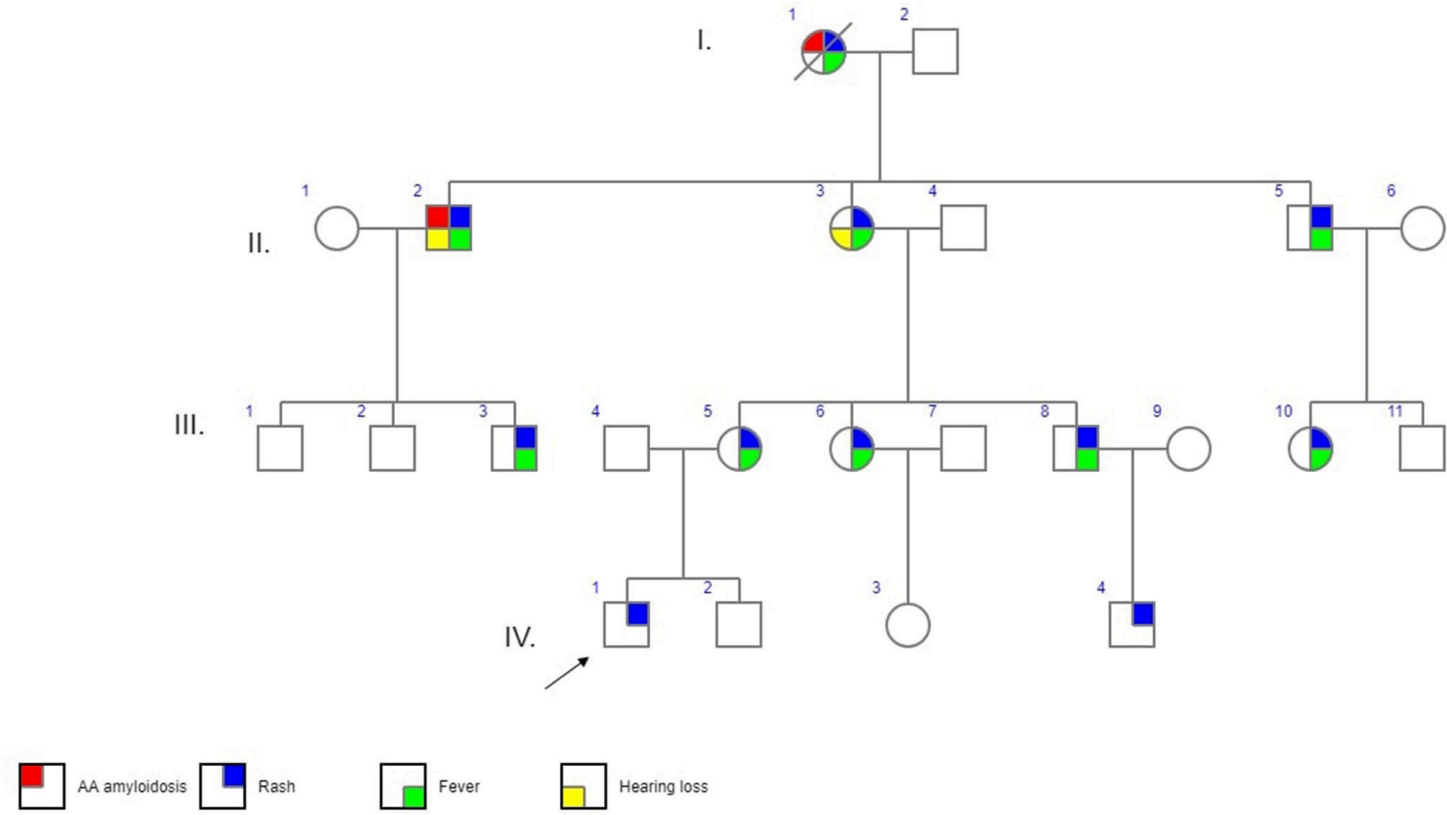
Family pedigree. I.-IV. – generations, 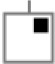 - affected male, 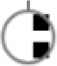 - affected female, 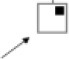 proband, 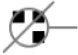 - deceased, probably affected (not genetically confirmed).

The first patient was a 3-years old boy (IV/1) suffering with recurrent flares of non-pruritic urticarial rash from 4 months of age. As his mother (III/5) reported similar long-term problems his primary care physician had organized a skin biopsy for her which showed non-specific changes. At the next referral to allergo-immunology an idea of a CAPS spectrum disorder was first raised and further supported by pediatric and adult rheumatology consultations. Genetic testing and further investigations were requested. While results were pending further consultations and communication within the family led to the discovery of other potentially affected family members who were offered appointments. Patient characteristics and their position in the family pedigree are shown in Table 1, Figure 1.

**Table 1:** Symptoms noticed before the diagnosis.

**Pt**	**Age at Dx (yrs)**	**F/M**	**Onset age (yrs)**	**Fever**	**Rash**	**Eyes**	**Arthritis**	**Hearing loss**	**Proteinuria**	**IL-1 blockade**
IV/4	1.5	M	0.2	–	+	–	–	–	–	–
IV/1	3.5	M	0.3	–	+	–	–	–	–	–
III/10	21	F	6	+	+	+	+	–	–	+
III/3	24	M	NA	+	+	+	+	–	–	–
III/8	27	M	7	+	+	+	+	–	–	+
III/6	31	F	3	+	+	+	+	–	–	+
III/5	31	F	2	+	+	+	+	–	–	+
II/5	43	M	NA	–	+	–	–	NA	NA	–
II/3	51	F	2	+	+	+	+	+	NA	+
II/2	52	M	4	+	+	+	+	+	+	+
I/1	52	F	NA	NA	+	+	+	+	+	Deceased

*Pt, Patient; Dx, Diagnosis; yrs, years; F, Female; M, Male; NA, not available. Eyes: conjunctivitis, uveitis, optic nerve atrophy, cataract, glaucoma, impaired vision*.

### Generation IV

Generation IV has 4 children (1.5–5 years), two of whom have been symptomatic. Both presented in early infancy with urticarial rash. Patient IV/1 is our proband. His rash has been increasing in intensity and frequency since about 2 years of age worsening in winter and in relation to stress (e.g., kindergarten). There have been no other symptoms of note, namely fevers, his inflammatory markers, audiometry and eye examination were normal. He has been followed-up closely by his local pediatric rheumatologist and remains off therapy for the time-being. His one-year old cousin (IV/4) presented a similar clinical picture with an absence of any other disease features apart from the rash and remains off-treatment.

### Generation III

Generation III consists of 8 individuals (21–33 years) with 5 of them affected. (Figure 1, Table 1). In 4 cases MWS was confirmed, one additional symptomatic family member has not yet become interested in specialized care (III/3). All patients reported symptoms since pre-school age triggered or aggravated, though not always, by cold weather. All of them perceived their disease as significantly interfering with their daily lives. Their initial work-up showed raised inflammatory parameters in all cases with no detectable organ involvement. During otherwise uneventful pregnancies prior to the diagnosis in patients III/5 and III/6 disease symptoms deteriorated in the former but improved in the latter. Therapy with anakinra 100 mg/day subcutaneously led to rapid improvement of all disease parameters within hours to days from the first injection in all of them. Only in patient III/5 the rash required 3 months of therapy to disappear. There have been no flares in any of the treated individuals and no need for anakinra dose escalation.

### Generation II

**Figure 2:**
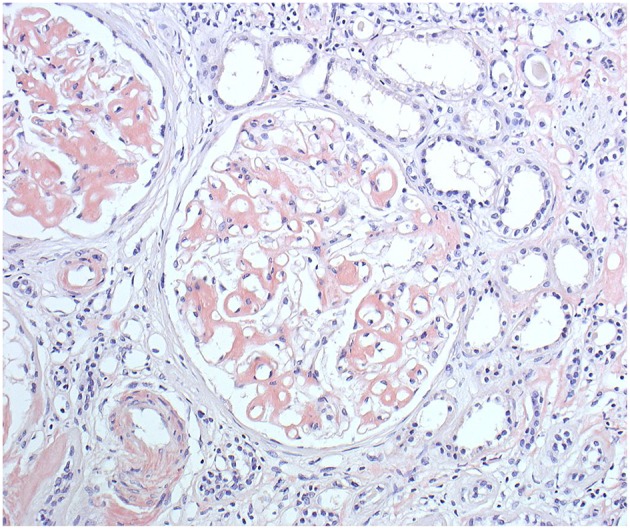
Patient II/2 renal biopsy. Congo red staining showing amyloid deposits in the glomeruli, arterioles and also in the interstitium around the peritubular capillaries. (Courtesy of Assoc. Prof. E. Honsova Ph.D, Clinical and Transplant Pathology Centre, Institute for Clinical and Experimental medicine, Prague, Czech Republic).

**Figure 3:**
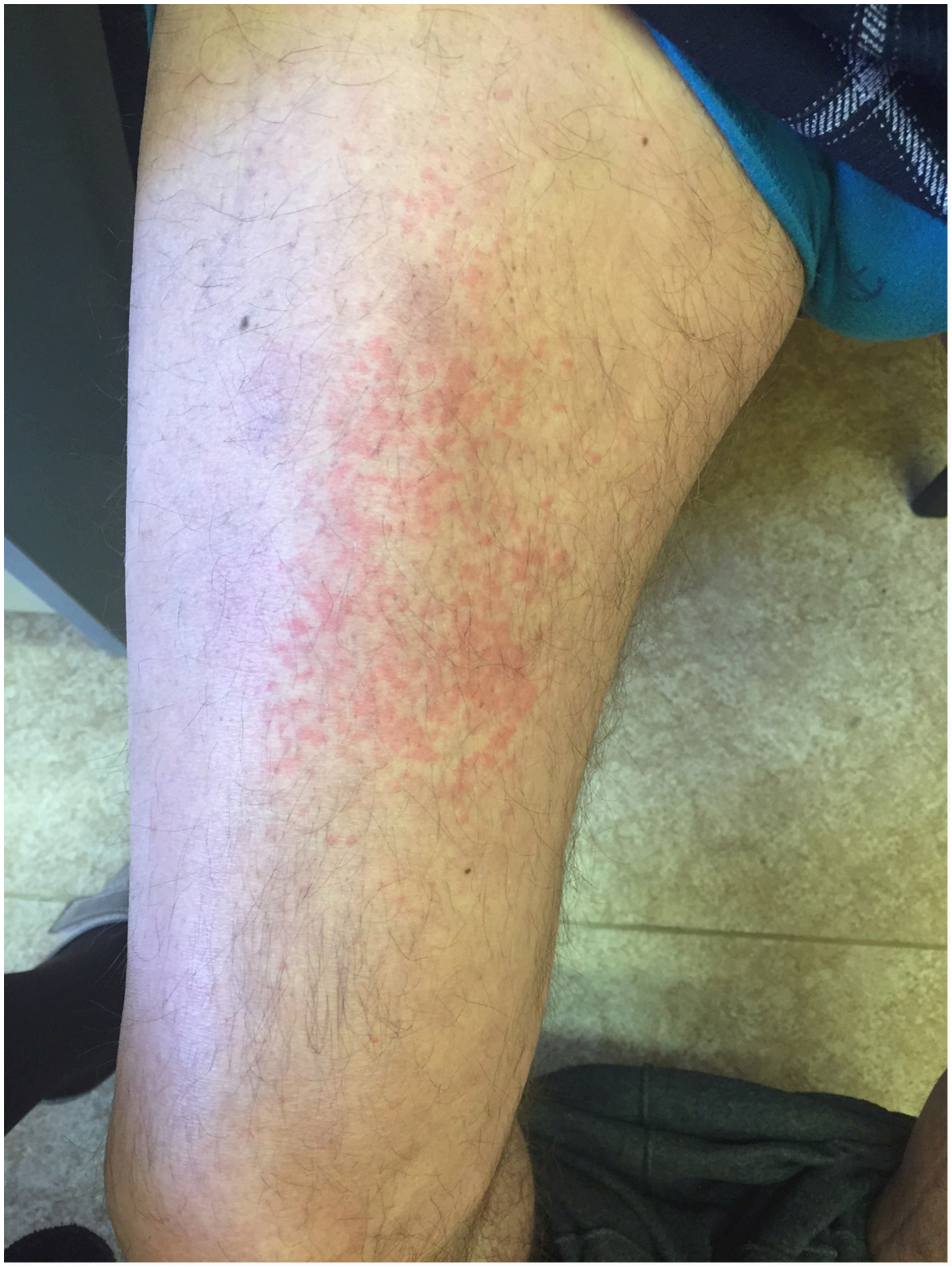
Skin rash in patient II/2.

Generation II has 3 siblings all affected. Patient II/2, a 53-years old proband's grand uncle, was originally referred to the local nephrology clinic for renal insufficiency with nephrotic proteinuria. As investigations in other family members were already running his renal biopsy was performed at the Czech center for amyloidosis. It confirmed presence of widespread AA amyloidosis (Figure 2). Being the oldest living affected family member he explained how the symptoms of fevers, rash (Figure 3), eye and joint problems have been so common in the family that people have become used to them. Unless the link to the renal failure and premature death of his mother was suggested he would not seek medical care. On top of the renal amyloidosis his medical workup revealed presence of sensorineural hearing loss (up to 30 dB in frequency of 6,000 Hz). Within 2 days of anakinra therapy all his systemic features disappeared. After 2 years of follow-up his renal functions remained stable with mild persistent proteinuria, audiometry showed no changes. Patient II/3, a 51-years old proband's grandmother, reported similar problems from her pre-school age. She remembered they were severe enough to prevent her attending school during cold months. Both physical and psychological stress as well as her 3 otherwise uncomplicated pregnancies led to further deterioration of these symptoms that reached their maximum from delivery throughout puerperium. Her medical work-up revealed mild elevation of inflammatory markers, normal renal parameters and presence of sensorineural hearing loss (from 30 to 60 dB according to increasing frequencies beginning in frequency of 500 Hz). Anakinra has led to the disappearance of all her symptoms and improvement of hearing by about 10–20 dB on repeated audiometry after 2 years of therapy.

Relatives of the patient II/5 (43 years old) reported him having mild daily rash which improved upon changing the work environment (from cold to completely warm conditions). He has not yet decided to seek medical assistance.

### Generation I

Proband's great-grandmother (I/1) died at 52 years of age (1994) from renal failure. Her medical records and genetic material were not available, therefore the history was based on the reports of her children (Generation II) who remembered her previous longstanding problems of fevers, rash and arthritis. Therefore, her retrospective diagnosis of MWS and renal amyloidosis was very likely despite the absence of genetic and histopathologic confirmation.

### Genetic Analysis

Genetic analysis of the *NLRP3* gene (exon 3) revealed heterozygous mutation c.1322C>T (rs121908146; c.1316C>T, NM_001243133.1) causing change of amino acids p. Ala441Val (p. Ala439Val, NM_001243133.1) in all affected and examined individuals. Patient II/2 has additional variant c.2113C>A (p. Gln705Lys) (rs35829419; c.2107C>A, p.Gln703Lys, NM_001243133.1) in the same exon. The frequency of the p. Gln705Lys is 3.8% according to GnomAD (http://gnomad.broadinstitute.org/) and is considered to be polymorphism (11).

### AIDAI

AIDAI diaries have been completed monthly by each treated adult patient (III/3, 5, 6, 8, 10 and II/2, 3). The pre-treatment AIDAI score ranged 20–77 points. Normal values were reached within 1-3 months by all patients and have remained so during the follow-up. The disease damage noticed at the time of diagnosis in patients II/2 and II/3 was 6 and 1 points of the ADDI score, respectively, and has not changed within the follow-up. No damage has been noticed in other affected individuals.

## Discussion

The study has identified 11 members of a single family with the clinical picture of MWS. The typical urticarial rash as a leading and the first symptom was present in all patients, similar to other reports (3, 12). Characteristics of CAPS patients reported to the Eurofever registry showed presence of rash in 97% of patients across the whole disease spectrum (3). Presence of fever was documented by 8/11 (72%) of our patients (in 84% in the Eurofever registry) (3). Elevated inflammatory markers were found in all adult patients but not in children. Hearing loss was detected only in 2 individuals (29%) from the oldest generation while in published series its frequency in MWS reached 85% (13). Comparison with published data is hampered by variable disease duration at the time of reports as well as by the presence of overlapping syndromes and genetic heterogeneity (5, 6, 14). It has been suggested that known CAPS phenotypes are not distinct but a part of the continuous disease spectrum (4, 15). Age at onset of hearing loss varied within as well as between individual affected families (16). In one Dutch family hearing impairment was detected in ages ranging from 4 to 25 years (16). Others reported this complication at 12, 16, and 9 years of age, respectively (14, 17, 18). In patients with the p.Val198Met or p.Ala439Val mutations, latter of which was detected in our cohort, progression of hearing loss was similar to that in healthy controls suggesting their mild impact (5). Relationship between *NLRP3* mutations and different trajectories of hearing loss suggested a mutation-specific risk (19). Stabilization or mild improvement in hearing loss in our patients was achieved with anakinra treatment.

Despite untreated disease AA amyloidosis developed only in individuals aged over 50 years in this family (I/1, II/2). In the Eurofever registry 5 out of 136 CAPS patients (4%) with AA amyloidosis aged 21 to 52 years were reported (3). Kuemmerle-Deschner found proteinuria in 10/13 patients (77%) (14). Lane et al. reported discovery of hereditary periodic fever in 50% of patients who presented to them with AA amyloidosis (20). It is anticipated that long-term IL-1 blockade may prevent development of this severe complication in other affected family members (7).

To our knowledge, effect of pregnancy on the course of CAPS and vice versa has not been reported. Only two women in the CAPS family described by Lequereé admitted disappearance of joint problems after pregnancy (1). Women in our family generally described an aggravation of their disease during each of the six pregnancies although severity of their symptoms was also affected by the season.

Affected individuals share heterozygous mutation p.Ala441Val. Genotype-phenotype correlations in CAPS have been studied by various groups. This mutation was detected in 10% of CAPS patients as reported by Levy et al. (3). Their phenotypic spectrum was milder (3) and it was negatively associated with neurological involvement (3). Its association with MWS/CINCA overlap and with FCAS and MWS was described (21). P.Ala441Val mutation was also noted in a patient with CAPS-related AA amyloidosis (3). The clinical significance of the Q705K (Q703K, p.Gln703Lys, NM_001243133.1) mutation carried by the only living patient with amyloidosis (II/2) remains uncertain as genotype of his deceased mother (I/1) is unknown and it has not been transmitted to any of his children. It is often classified as a functional polymorphism (3) or as a variant of unknown significance (allele frequency of up to 5%) (22). However, one paper connected it with an increased release of proinflammatory cytokines (11).

We have reported striking phenotypic differences between individual generations. Due to the simultaneous disease detection in untreated individuals of different age the natural disease course was described in the real-time. It ranged from the mild, localized features in early childhood through typical systemic, skin, ocular, and joint symptoms in young adults to evolution of hearing loss and renal amyloidosis in older individuals. The history of fevers varied in different patients, though it was at some point reported by all except children. Such a varying clinical presentation may also suggest possible involvement of other factors affecting phenotype expression like modifier genes (14, 23) or yet unknown age-related mechanisms. One mutation can cause different phenotypes combining features of FCAS and MWS within one family (15, 24) as well as between families (23).

Disease symptoms had disappeared within hours to days from starting anakinra in all patients, which is in line with the published series (25). Improvement of hearing loss and amyloidosis was shown in individual patients when treatment was introduced early (12, 26). Rynne et al described reversibility of hearing loss in a 59 years-old woman with MWS but younger age might offer a window of opportunity for reversing hearing loss (26, 27). Our findings so far confirmed mild improvement or stabilization of hearing loss after 2 years of therapy in both affected patients (II/2, II/3). Question about the optimal timing of initiating treatment in the youngest, oligo-symptomatic patients in order to prevent development of irreversible damage remains unanswered. Common sense suggests that as long as systemic inflammation is absent both clinically (no fevers) and in blood tests (normal SAA) therapeutic delay should not be harmful. Nevertheless, this approach may not be applicable in families carrying different CAPS mutations. In Europe two IL1 blockers are registered for the treatment of CAPS. Our choice of daily anakinra over 8-weekly canakinumab in adult patients had two main reasons: its dose is more easily adjustable and its price is significantly lower. Once affected children require treatment, canakinumab will be the first option for compliance reasons.

All patients have got used to regular completing of AIDAI questionnaires. Decline in AIDAI values mirrored their clinical and laboratory improvement. Although disease damage as reflected by ADDI was detected only in patients from the oldest generation, it has not deteriorated over the relatively short follow-up period. Indeed, use of validated assessments including the abovementioned instruments for regular evaluation of disease activity and damage has been suggested in the recently published recommendations for the management of autoinflammatory diseases (28).

Several extended families with multiple members affected with MWS, FCAS or their overlaps were reported (1, 8, 12, 14, 16, 21, 29). Nevertheless, none of them presented combination of clinical and laboratory characteristics of patients in different generations prior to the treatment nor the follow-up including standard patient-reported outcomes.

## Conclusions

Retrospective and cross-sectional analysis of multiple members of one large family affected with MWS has shown that amount and severity of symptoms gradually increased with age toward distinct phenotypes typical for members of each of the four generations. IL-1 blockade with anakinra induced remission of active inflammatory features in all treated individuals. Without linking pediatric and the oldest family member histories together adult patients from this kindred would not seek medical care for their long-term symptoms as they felt these were part of their inevitable heritage. This report illustrates importance of the high degree of suspicion as well as importance of the close collaboration among pediatric and adult specialists in order to establish timely diagnosis and initiate appropriate investigations and treatment.

## Ethics Statement

This study was carried out in accordance with the recommendations of the Ethics committee of the General University Hospital with written informed consent from all subjects. All subjects gave written informed consent in accordance with the Declaration of Helsinki. The protocol was approved by the Ethics committee of the General University Hospital in Prague.

## Author Contributions

ŠF and PD coordinated and performed most of the clinical analysis and drafted the manuscript. JF, EHl, EHo, EJ, and LP contributed to clinical observations and patients follow-up. MT and KB performed and interpreted genetic data. All authors contributed to conception, analysis and interpretation of data and participated in revising the article and gave final approval of the version to be submitted.

### Conflict of Interest Statement

The authors declare that the research was conducted in the absence of any commercial or financial relationships that could be construed as a potential conflict of interest.
